# Can Habits Impede Creativity by Inducing Fixation?

**DOI:** 10.3389/fpsyg.2021.683024

**Published:** 2021-10-13

**Authors:** Paula Ibáñez de Aldecoa, Sanne de Wit, Sabine Tebbich

**Affiliations:** ^1^Department of Behavioural and Cognitive Biology, University of Vienna, Vienna, Austria; ^2^Department of Clinical Psychology, University of Amsterdam, Amsterdam, Netherlands

**Keywords:** creativity, habits, fixation, divergent thinking, problem-solving, frequency of use

## Abstract

In a competitive and ever-changing world, the ability to generate outstanding ideas is crucial. However, this process can be impeded by factors such as fixation on ideas that emerged through prior experience. The aim of the present study was to shed light on the fixating effect of habits on creativity. To this end, healthy young adults were asked to generate alternative uses for items that differed in their frequency of use in the Alternative Uses Task (a standardized test for divergent thinking). We predicted that frequent past use of an item would lead to the formation of stimulus-response associations between the item and its most frequent use(s) and thereby hinder idea generation. Indeed, individuals were less flexible (but more fluent) in generating ideas for frequently used items than for unknown items. Additionally, we found that subjective automaticity of idea generation was negatively related with flexibility. Finally, we investigated whether individual differences in general habit tendency influence creativity, by relating performance on the Slips-of-Action task (an outcome devaluation paradigm extensively used in habit research) to performance on the Alternative Uses Task, the Candle Problem (a classic convergent thinking task) and two puzzles (non-conventional problem-solving tasks). While we did not find a significant relationship between habit tendency and the Alternative Uses Task or the Candle Problem scores, the tendency to rely on habits predicted probability to succeed and latency to solve one of the puzzles: less habit-prone participants were more likely to solve it and to do so faster. In conclusion, our study provides evidence for the notion that habits can negatively impact creativity and opens promising future avenues of research in this field.

## Introduction

In a rapidly changing world, which forces us to confront new challenges every day, individuals who can generate innovative ideas and solutions have a distinct advantage over less creative thinkers. Creativity, which can be defined as the sequence of processes that culminate in the production of something new and worthwhile (Sternberg, 2011; Runco and Jaeger, 2012), is a key resource in our changeable world, as it enables one to profit from unexploited opportunities and to cope with novel and unforeseen situations (Soriano de Alencar, 2012). However, to maximize the benefits of creative potential, one must first overcome the factors that hinder it, such as moving past old ideas “inside the box” (Storm and Angello, 2010). These “old ideas” can induce fixation, which refers to the difficulty in fully identifying the possible ways to use an object (i.e., its affordances) beyond its familiar uses (Gibson, 1977), due to habitual experience with the object in a particular context (Duncker, 1945; Flavell et al., 1958). Indeed, systematically approaching problems following previously learned examples is a common impediment to the creative process (Crilly, 2015, 2019; Ho and Shu, 2018): when one solution for a given problem is known, the subsequently generated solutions tend to replicate features of the known solution (Jansson and Smith, 1991). If a particular response (R) or solution (e.g., drawing) is repeatedly performed in the presence of consistent contextual stimuli (S) or a consistent object (e.g., a pencil), a S-R association (and eventually a habit) is formed, such that, on future occasions, the stimulus can directly prime the behavior through the S-R association (e.g., pencil-draw; de Wit and Dickinson, 2009; de Wit et al., 2018). This strengthened S-R association and the ensuing behavior or thought pattern are known as a habit. Habits could hamper creativity because mental activation of old solutions can impair the consideration of novel ideas or responses (e.g., pencil-drumstick), ultimately leading to a loss of flexibility in behavioral control (Gillan et al., 2011; de Wit et al., 2012a).

To investigate the role of habits and familiarity in fixation we used the Alternative Uses Task (AUT), a classical divergent thinking paradigm where participants have to come up with alternative uses for objects (adapted from Guilford, 1967). Here, idea generation was compared for items that differed in their frequency of use by using frequently used, rarely used, and unknown items. To control for familiarity (German and Barrett, 2005), we presented objects for which participants have differing frequencies of past use (e.g., a coffee cup versus an unfamiliar object from a different culture) and investigated whether AUT performance was affected by habits. We predicted a stronger effect for frequently used items, as they would presumably elicit persistent mental activation of their most common usage(s) through well-established S-R associations. In contrast, the effect for unknown items was expected to be low or non-existent due to the absence of opportunities to form S-R associations. We expected these patterns to lead to higher *flexibility* (number of metacategories of use cited, which reflects the variety of responses) in idea generation for unknown items compared to frequent items. On the other hand, we expected higher *fluency* (number of adequate ideas provided) for frequent items, since recalling existing associations should enable faster identification of an item’s affordances. We omitted the *originality* index from our analysis of creativity because we considered that judging originality for unknown items relative to familiar items would be prone to yield inaccurate estimates, as many ideas for unknown items may seem more original than they actually are simply because one ignores what the most common use for those items is. We therefore focused our analysis on fluency and flexibility, for which we had clear predictions. Additionally, we measured subjective automaticity of idea generation (hereafter referred to as *automaticity*) by asking participants how automatically they thought of the most common use for each item. We predicted that usages for frequent items would be thought of more automatically due to pre-existing S-R associations, and that this would, in turn, negatively impact flexibility. We did not have, however, a strong prediction concerning its effect on fluency.

Interestingly, recent studies in the research field of habits suggest that people differ “habit tendency,” or the degree to which they tend to rely on habitual versus flexible, goal-directed control (Watson and de Wit, 2018). This is reflected in interindividual variability in the ability to suppress cue-triggered, learned responses when the outcome has been devalued and is therefore no longer desirable, as measured with outcome-devaluation paradigms. Impaired performance on these paradigms is interpreted as an overreliance on habits that could be due to strong habit formation or to weak goal-directed control, or indeed a combination of the two. If habits underlie reliance on pre-formed ideas, habit-prone individuals may be more vulnerable to fixation, either because they require fewer stimulus repetitions to form strong S-R associations, or because they have a weaker capacity to act in a flexible, goal-directed manner. To investigate this, we assessed whether individual differences in habit reliance are related to divergent thinking. Habit tendency was evaluated with the Slips-of-Action Task (SOAT; de Wit et al., 2012b; Sjoerds et al., 2016; Watson and de Wit, 2018). Here, “slips of action” toward devalued outcomes are interpreted as evidence for habit tendency. We related habit tendency to divergent thinking (performance on the AUT) and predicted that habit-prone individuals would rely on previous knowledge rather than on the generation of new ideas when solving a problem or assessing object or task affordances. As a result, they should show lower flexibility for frequently used items than participants who were less prone to rely on S-R associations. In other words, we predicted that habit-prone individuals are more vulnerable to fixation, while “out-of-the-box thinkers” are relatively goal-directed (Valentin et al., 2007; Morris et al., 2016).

Participants were also tested in a well-established convergent thinking paradigm known as the Candle Problem (Duncker, 1945). This task entails a fixation component, since solving the problem requires one to overcome fixation on the normal functional uses of an object. Individual performance on this task was related to performance on the SOAT. We predicted that habit-prone individuals should perform more poorly due to their difficulty in overcoming fixation. Finally, we explored whether individual performance on the SOAT correlated with the probability of solving a less conventional convergent thinking task, namely puzzle games. Despite often being overlooked in adult studies, they have been used to test convergent thinking in children (Muller and Perlmutter, 1985). Puzzles are problem-solving tasks that require the interplay of working memory, inhibitory control and insight, and are advantageous because they do not require the use of language. In addition, in contrast to well-established convergent thinking tasks like the Candle Problem, participants do not have pre-formed S-R associations regarding the specific items involved but they do have to overcome a more general pre-existing knowledge (e.g., the habit of building in horizontal layers).

Creative ideas help us navigate the complexities of our everyday lives but producing them requires leaving old habits behind (McWilliam and Haukka, 2008). Our study aims to determine the role of habits and automaticity in creative thinking. To this end, we bridged the gap between research in the fields of creativity and habits by combining experimental paradigms traditionally used in these disciplines. To our knowledge, this study is the first to relate familiarity, subjective automaticity, and habit tendency to the two major dimensions of creativity (Guilford, 1967), namely divergent thinking (characterized by spontaneity that promotes a burst of ideas) and convergent thinking (which entails recalling an existing solution). Understanding the factors that make some individuals more prone to fixation than others could help to improve creative performance.

## Materials and Methods

### Ethics Statement

All procedures performed were in accordance with the ethical standards of the institutional and national research committee and with the 1964 Helsinki Declaration and its later amendments. The study was approved by the University of Amsterdam Faculty of Social and Behavioral Sciences Ethics Review Board (Ref. No.: 2018-CP-8939). Informed consent for participation and data publication was obtained from all individual participants included in the study.

### Participants

Eighty-five students (61 females, 24 males) aged 18–30 (average 23.29 years old, *SD* = 4.08) participated in this study at the University of Amsterdam (UvA). Forty-three of the participants majored in psychology, 37 in other social sciences and the rest in life sciences. The inclusion criteria were: (1) not having participated in a similar study before, (2) being a Dutch native speaker, and (3) being fluent in English. Enrollment in the study was done on a voluntary basis, and aborting was possible at any time.

### Procedure

The experiment took place at the UvA from March to June 2018. An experimenter remained in a corner of the room during testing sessions and did not interfere with the subject’s performance or provide any feedback. The study consisted of three computer tasks and two puzzles. The order of presentation of the different tasks was not randomized across participants. Participants were allowed to 90 min to complete all tasks, and short breaks between tasks were provided when needed. Before testing, participants completed a general questionnaire to gather demographic information and personal data, which remained confidential. Participants were identified by an anonymous code for the purposes of data collection and analysis, and they were asked to read the information brochure of the study prior to testing. At the end of the test session, participants were debriefed about the purpose of the experiment.

#### Task I. Alternative Uses Task

Divergent thinking was assessed by the Alternative Uses Task (AUT; Guilford, 1967), which has been extensively used to measure appropriate and novel responses to an open-ended task. The computer task was programmed with Presentation version 20.1 (Neurobehavioral Systems, built on 12.04.17) by the Research Support Unit of the UvA. Six different items belonging to three categories of use (two items per category) were selected. The use categories were: frequently used, *freq* (cup and pencil), rarely used, *rare* (brick and rope), and unknown items, *unk* (bamboo tea whisk and ancient medical instrument). Frequency of use was established based on a previous survey (see Supplementary Table 3). Outcome responses were divided into three groups according to their frequency of use, and two objects from each group category were picked at random by using the *sample* function in R. These items constituted the target stimuli presented in the AUT. Frequency of use was confirmed afterward through follow-up questions to the participants: 87.65% of them reported that they used the frequent items at least once a week, whereas 94.12% reported that they had never used the unknown items before. Presentation of these six items was equally distributed into two blocks of three items each and order of presentation was counterbalanced across participants (order 1: freq-rare-unk; order 2: unk-freq-rare; order 3: rare-unk-freq). In contrast to previous studies in which only the names of items were provided (see Snyder et al., 2004; Silvia et al., 2008; Boot et al., 2017 for some examples), stimuli were presented as images to ensure a homogeneous representation across participants. As in similar, recent studies (Forthmann et al., 2016; Boot et al., 2017), each item image was presented separately for a total of 120 s with a fixed inter-stimulus interval of 5 s. The image remained on the computer screen during the entire trial, while participants typed their answers in the blank space provided below. Instructions were presented in Dutch, as well as answers provided by the participants, which were later translated into English by a bilingual assistant. In accordance with the two distinct hallmarks of creativity, namely usefulness and newness (Amabile and Mueller, 2008; Runco and Jaeger, 2012), participants were encouraged to be as creative as possible, but to provide valid (i.e., appropriate) answers. They were asked to try to think of as many novel, alternative ways of using each item as possible and were explicitly instructed to focus on uses they considered less conventional and to avoid listing the most common ones. The list of uses already provided by a participant for a given item did not remain visible on the screen as new responses were typed in. After completing the task, participants were asked to report the frequency with which they personally used each item (5-point Likert scale: 1 = never, 5 = every day), and how automatically they thought of the most common use for each item (5-point Likert scale: 1 = not automatically at all, 5 = highly automatically).

Validity, often referred to as appropriateness (Chamorro-Premuzic, 2006) or adequateness (Reiter-Palmon et al., 2019), is a commonly applied inclusion criterion in AUT scoring. Hence, ideas were first verified in terms of appropriateness, so that only appropriate ideas were considered for scoring. We disregarded the uses that were not applicable to the item given its affordances (i.e., any use that made no sense given the features of the item, or redundant answers). An example of inappropriate use for a “pencil” was “tying objects together,” due to the impossibility of this action. Once responses deemed inappropriate were discarded, the remaining answers were scored by two independent, trained coders, who were blind to all hypotheses and to all predictors and their levels. Fluency and flexibility ratings were averaged for all objects across the two coders, yielding a high interrater reliability (all ICCs > 0.80, all *p*-values < 0.001). Responses were scored using an approach developed from the relative frequency of ideas criteria (Runco et al., 1987) and the objective scoring system (Snyder et al., 2004). Both methods ensure that the uses suggested in radically different categories are worth more points. In our study, however, we avoided assigning weights to each individual idea and then summing them. Instead, we first grouped ideas into distinct categories according to shared functionality, and then used these categories to calculate a flexibility index that was independent of fluency. This creativity assessment method avoids the confounding with fluency (Silvia et al., 2008), as it yields distinct estimates for both quality and quantity. Points were assigned for the two indexes given below and scores for both items of the same type (pencil-cup; brick-rope; whisk-medical instrument) were averaged:

(i) Fluency, *flu* (quantity of ideas): the sum of total valid answers per participant for each type of item. This value could potentially range from 0 to infinity.
(ii) Flexibility, *flex* (quality of ideas): calculation of this index required grouping usage responses according to functional similarity and conceptual relatedness into metacategories. The pooled responses from all participants were used to determine the global maximum number of metacategories cited for each item. The flexibility index was then calculated for a given item and participant, by dividing the number of participant’s metacategories for that item by the number of global metacategories for the item, rendering a ratio ranging from 0 (least flexible) to 1 (most flexible).

#### Task II. Slips-of-Action Task

**FIGURE 1:**
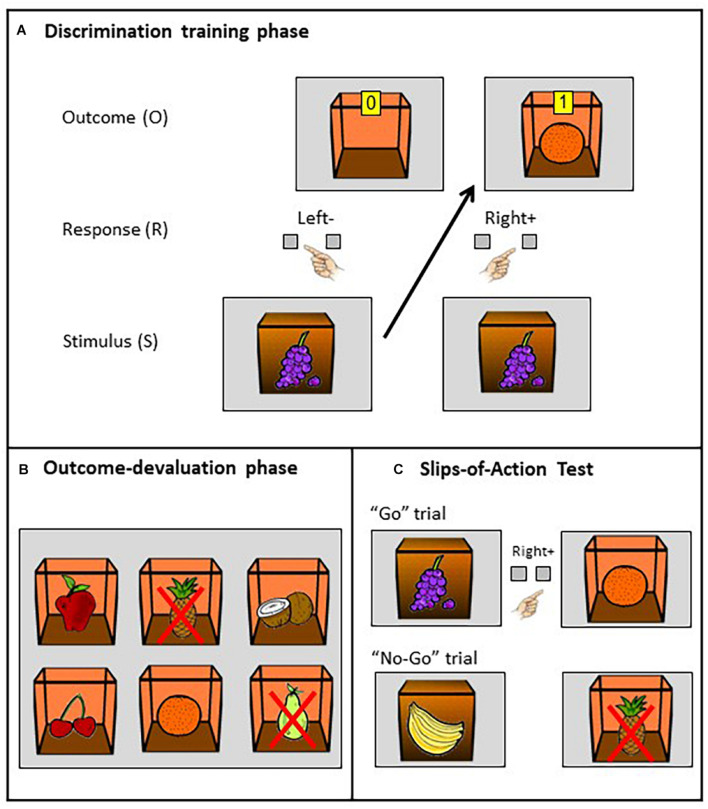
Example of a trial in the SOAT. Explanation of SOAT based on one example configuration. **(A)** Discrimination training phase: pressing the right key (response, R) when a grape cue (stimulus, S) was presented on the closed box led to successfully opening it, earning points (marked on a yellow square with the number 1) and obtaining an orange (outcome, O) as a reward. On the contrary, pressing the left key led to no points (0 on the yellow square) and an empty reward box. Acoustic and visual feedback was given for correct and wrong answers, which enabled participants to learn the S-R-O associations. **(B)** Outcome devaluation phase: at the beginning of each block of trials, all the existing fruit outcomes (six in total) were briefly shown together, with two of them being devalued and therefore no longer worthy of points (crossed out in red; in this example, pineapple on top row center and pear on bottom row right), indicating that the response leading to these outcomes will now result in subtraction of points; the other four outcomes (apple, coconut, cherry, orange) remained non-devalued. **(C)** Slips-of-Action Test: fruit stimuli on closed boxes were rapidly shown one by one. Participants had to press the correct key when the corresponding outcome had not been devalued. The accurate response in this example would be pressing the right key to obtain an orange outcome upon presentation of a grape stimulus (“go” trial). However, upon presentation of the banana stimulus (linked to pineapple: devalued outcome), participants should withhold their response (“no-go” trial). No feedback was given at this stage. Image adapted from de Wit et al. (2012b) and Sjoerds et al. (2016) (obtained with permission).

Habit tendency was measured by a simplified version of the SOAT. This computerized paradigm has been broadly used to study the balance between goal-directed and habitual action control and permitted us to relate inter-individual variation in habit tendency to measures of divergent and convergent thinking. For a detailed description of the task, the reader is referred to Worbe et al. (2015). After a simplified demonstration of the task, in phase one (discrimination training phase; see Figure 1A) participants were instructed to earn as many points as possible by collecting fruits from inside a box on the screen (i.e., the outcomes; O) by pressing either the right (“M”) or left (“Z”) key for each fruit stimulus that appeared outside of the box (i.e., the discriminative stimuli; S). While pressing the correct key opened the box and revealed a fruit and points inside, pressing the incorrect key led to an empty box and no points. The purpose was to learn which key to press by using six different fruit icons on the front of the box as cues. Additionally, more points were earned if the response was performed faster. Participants had to learn by trial and error which key to press for each stimulus and which of the six fruit outcomes could be found inside the boxes. They were instructed to pay attention to the outcomes, as, in order to be successful in the second phase of the task, they needed to recall the S-R-O combinations from the first phase and gather some types of fruit but not others. There were six different combinations of fruit pairs (S-O) and associated R; the fruits used as cues were different from the fruits used as outcomes. These S-R-O combinations were permutated across participants, but remained consistent across trials for a given participant. This phase consisted of eight blocks, each with a duration of 7–8 min (self-paced), and a total of 84 trials.

At the start of the second phase (outcome devaluation phase or *slips-of-action test;* see Figure 1B), some of the fruit outcomes were devalued, meaning that pressing a key upon presentation of the corresponding stimulus would lead to a loss of points. Subsequently, participants were presented with the discriminative stimuli again in rapid succession (see Figure 1C). The least habit-prone individuals were expected to respond only to stimuli signaling non-devalued outcomes but withhold their learned response for devalued outcomes. On the contrary, participants who formed strong S-R habits (or participants with weak goal-directed control) should be more vulnerable to commit errors (to suffer a “slip-of-action”) in the trials were outcomes were devalued. This phase consisted of 108 trials, whereby each trial unit was composed of an instruction (5 s) and the test stimulus presentation (1 s), with a fixed inter-trial interval (1.5 s).

The score for this task, referred to as *difference score*, was calculated by subtracting the percentage of responses to stimuli signaling devalued outcomes (i.e., *no-go* trials) from the percentage of responses to stimuli signaling valued outcomes (i.e., *go* trials). This generated scores from 0 (most habit-prone) to 100 (least habit-prone). Goal-directed control should result in successful suppression of a learned response and consequently to a higher difference score, while habitual control should lead to more errors due to perseverance and, hence, to a lower score. A score of a 100 would result from a response rate of 100% to boxes with valued outcomes (*go* trials) and a 0% response rate to boxes with devalued outcomes (*no-go* trials), indicating perfect goal-directed control.

#### Task III. the Candle Problem

This standardized task (Duncker, 1945) is typically used to evaluate the influence of fixation on problem-solving abilities, since it requires the subject to overcome fixation on the conventional use of an object or one of its parts, moving beyond automatic behaviors (Hennessey and Stathis, 2015). For this task, a drawing depicting a candle, a pack of matches and a box of pushpins was shown to participants on the computer screen. They were then asked to explain, by typing in their answer, how they would affix the candle to a wall using the objects provided in the picture, so that the melting wax would not drip onto the surface below. As in previous studies (McCaffrey, 2012), participants were allowed up to 8 min (480 s) to complete this task. A solution was considered correct when participants (a) successfully attached the candle to the wall, in accordance with the instructions given, and (b) overcame fixation by realizing that the pushpin box was not only a container but could also be used as a platform to hold the candle upright. Arrival at this solution required participants to escape fixation on the most common affordance of a familiar item (that of the box as a container) to enable attendance to its other potential uses (e.g., as a platform). We measured (a) success (score = 1 point) or failure (score = 0 points) to solve the problem, and (b) latency to success (a value of 0 was assigned to participants who did not solve the problem or gave up). The score for solvers was calculated using the formula: 1 − (duration to success in seconds/480). This granted a higher score to participants who solved the task faster. Data for 16 participants was not usable, since they declared that they already knew the problem and its correct solution beforehand.

#### Task IV. Puzzles

**FIGURE 2:**
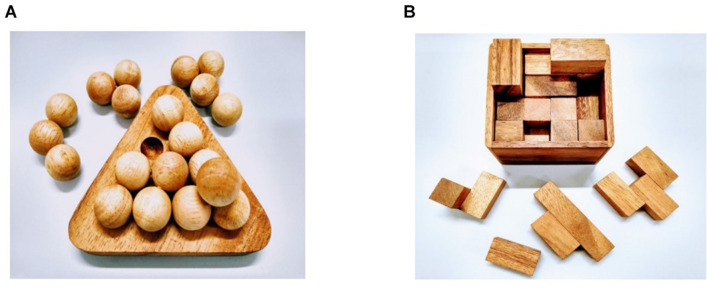
Puzzles used for Task IV. **(A)** Pyramid, consisting of 6 pieces, each one composed of a differing number of spheres and spatial configuration; **(B)** Cube, consisting of angular pieces of different shapes. The cube volume was 6.8 cm^3^ when assembled.

To evaluate an additional dimension of convergent thinking, namely operant problem-solving, we used two brain teasers: a pyramid and a cube puzzle (Dilemma Games^1^; see Figure 2). These puzzles required participants to assemble a number of wooden pieces into a successful configuration in order to build a structure and solve the problem. Participants were allowed to try to solve each puzzle for a maximum of 5 min (300 s). Participants were told there was a time limit to solve them, but the time limit was unspecified. They were also informed that they could give up before time was up. We measured (a) success (=1 point) or failure (=0 points) to solve each puzzle within the time limit, and (b) latency to success (a value of 0 was assigned to participants who did not solve the problem or gave up). The score for solvers was calculated using the formula: 1 − (duration to success in seconds/300). Additionally, we measured the ability to overcome fixation in order to attain success within the time limit (see Table 1).

**TABLE 1:** Fixation scoring for both types of puzzles on Task IV.

Puzzle	Not overcoming fixation (=0)	Overcoming fixation (=1)
Pyramid puzzle	Not using balls in vertical	Using balls in vertical
Cube puzzle	Building cube inside box	Building cube outside box

*For the pyramid puzzle, the only successful way to combine the pieces was by placing some of them upright (in vertical position).*

*Only by overcoming the initial tendency to assemble them horizontally could the problem be solved.*

*For the cube, it was advantageous, though not strictly necessary, to assemble the pieces outside the box.*

### Statistical Analysis

We tested our hypotheses using model fitting procedures, adapting the model type appropriately to the response variable type. We used linear mixed-effects models for the numeric outcome variables (*fluency*, *flexibility*, *automaticity*, and *latency*) and logistic mixed-effects models for the binary outcome variables (*solved*, *fixation*). Each model included the main effects of one to four predictor variables (*type of object*, *habit*, *order of presentation*, and *automaticity*) on a single (numeric or binary) response variable. In addition, we included *participant* as a random effect (random intercept) in each model (see Supplementary Table 1 for a detailed explanation of these variables and Table 2 for details on the fitted models). In Task I (AUT), the influence of *type of object*, *habit*, and *order of presentation* on *automaticity, fluency*, and *flexibility* was assessed (Models 1, 2, and 3, respectively). In these three models, unknown items were considered as control stimuli, as we assumed participants should not have pre-formed associations to such items. In Task III (Candle Problem) and Task IV (puzzles), instead of re-using the same dataset, we created three subsets to test each hypothesis separately, namely “pyramid set” (Models 4a, 5a, 6a), “cube set” (Models 4b, 5b, 6b), and “candle set” (Models 4c, 5c), respectively. Each of these subsets, therefore, contained the data for one of the corresponding tasks (number of participants was *N* = 85 for the pyramid and cube subsets, and *N* = 69 for the candle set; note that, in the case of the Candle Problem, 16 participants declared that they already knew the task and its solution (data not shown; discarded for this analysis), reducing the sample size for this task from 85 to 69). With these subsets, we then run separate models to evaluate the effect of *habit* on *ability to solve* (Models 4a,b,c), *latency* (Models 5a,b,c), and *fixation* (Models 6a,b). Additionally, we performed three independent *t*-tests (one for each type of problem) to compute differences in habit tendency between solvers and non-solvers. As we did not hypothesize about the effect of age and sex, these variables were not included as predictors in any of the models.

**TABLE 2:** Solvers and non-solvers for each type of problem in Tasks III and IV.

Task	Solvers	Non-solvers	Gave up
Candle Problem	56 (81.16%)	13 (18.84%)	0 (0.00%)
Pyramid puzzle	28 (32.94%)	50 (58.83%)	7 (8.23%)
Cube puzzle	20 (23.53%)	63 (74.12%)	2 (2.35%)

*Participants who solved, did not solve, or gave up on each of the problems presented for Tasks III and IV (percentages in brackets).*

*For the Candle Problem, 16 participants already knew the problem and its solution before taking part in the experiment (data not shown; discarded for this analysis), reducing the sample size from the initial 85 participants to 69 participants for this task.*

Prior to fitting all models, we inspected the symmetry of distribution of all quantitative predictor and response variables. A normality check of residuals (Q-Q plot; Field, 2005) and a plot against fitted values (Quinn and Keough, 2002) indicated no deviations from these assumptions. The statistical analysis was conducted with RStudio (version 3.6.1; R Core Team, 2019), using the package lme4 (version 1.1-21; Bates et al., 2015), function *lmer* for the linear mixed models, and function *glmer* for logistic regression, as well as the package ggplot2 (version 3.3.0; Wickham, 2016) for plotting the results. We determined 95% confidence intervals of fitted values using the function *confint* of the package *base* for the lmer models (Models 1, 2, 3, and 5a,b,c of Supplementary Table 2) and a custom-built function for glmer models (Models 4a,b,c and 6a,b of Supplementary Table 2). Prior to data collection, statistical power (α = 0.05; effect size = 0.75; power = 0.95) was calculated with G^∗^Power 3.1.9.2 (Faul et al., 2007), indicating 80 ± 4 as an adequate sample size. Effect size determination (Kelley and Preacher, 2012) was based on similar previous studies (Friedel et al., 2014; Sjoerds et al., 2016).

## Results

### Divergent Thinking: Alternative Uses Task

#### Automaticity

**FIGURE 3:**
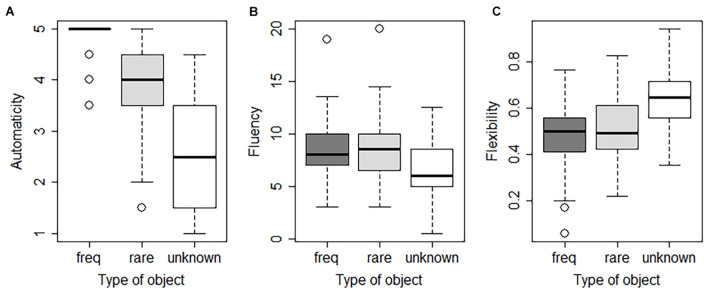
Automaticity, fluency, and flexibility of responses generated for the AUT. **(A)** Self-reported automaticity (range: 1–5) for frequent (freq; in dark gray), rare (rare, in gray) and unknown (unknown, in white) in Task I. **(B)** Number of ideas generated (fluency; range: 0–20). **(C)** Number of categories cited (flexibility; ratio range: 0–1). Box plots display the median, lower, and upper quartiles, with whiskers representing values within 1.5 times the interquartile range. The individual dots represent outliers.

Self-reported automaticity scores (mean ± SD) were 4.91 ± 0.26 for frequent items, 3.92 ± 0.79 for rare items, and 2.60 ± 1.08 for unknown items. To further examine the factors that contribute to automaticity in the AUT, we fitted a linear mixed-effects model to the numeric outcome variable *automaticity* as a function of *type of object* and *habit*, with *participant* as a random effect. While no effect of *habit* (mean score ± *SD* = 67.49 ± 19.14) was found (*F*_(__1_,_82__)_ = 0.016; *p* = 0.9), self-reported automaticity differed between object types (see Figure 3A; see Model 1 of Supplementary Table 2): participants reported that they thought more automatically of the most common use for an item when it was frequently used. In particular, automaticity was 53% higher for rare items and 92% higher for frequent items (*F*_(__2_,_168__)_ = 193.772; both *p-*values < 0.001) compared to unknown items.

#### Fluency

We fitted a linear mixed-effects model to the numeric outcome variable *fluency* as a function of *type of object*, *automaticity*, *order of presentation*, and *habit*, with *participant* as a random effect (see Model 2 of Supplementary Table 2). On average, the number of ideas generated for frequent and rare items was significantly higher than for unknown items (29 and 30% higher, respectively; *F*_(__2_,_168__)_ = 62.179; both *p*-values < 0.001; see Figure 3B). Order of presentation did not have a significant effect on fluency (*F*_(__1_,_82__)_ = 0.554; *p* = 0.452), meaning that seeing the frequent, rare, or unknown item first did not affect the number of ideas generated. Individual differences in habit tendency, as measured by the SOAT, did not significantly correlate with fluency (*F*_(__1_,_82__)_ = 0.216; *p* = 0.637). There was no significant effect of automaticity on fluency (*F*_(__1_,_184__)_ = 1.208; *p* = 0.273).

#### Flexibility

**FIGURE 4:**
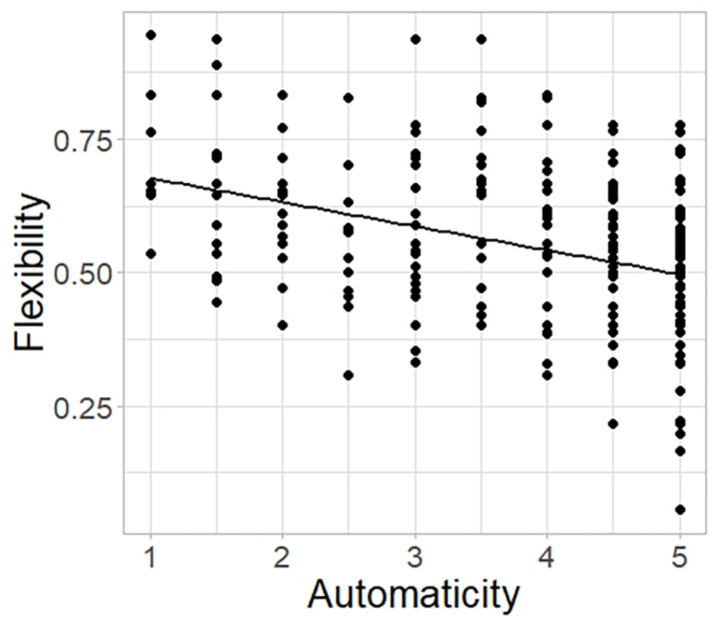
Effect of self-reported automaticity on flexibility in the AUT. Overall effect of self-reported automaticity (range: 1–5) on flexibility (range: 0–1; *p* < 0.001) in the Alternative Uses Task (AUT). The regression line depicts the prediction made by the model. Data points are spread out to avoid overlapping, as participants were allowed to assign the same automaticity score to all types of objects equally.

We fitted a linear mixed-effects model to the numeric outcome variable *flexibility* as a function of *type of object*, *automaticity, order of presentation*, and *habit*, with *participant* as a random effect (see Model 3 of Supplementary Table 2). Flexibility depended on type of object and was on average 21.7% lower for rare items and 27.3% lower for frequent items (*F*_(__2_,_168__)_ = 39.777; both *p*-values < 0.001; see Figure 3C) compared to unknown items. We found a significant negative effect of automaticity on flexibility, with a decrease of 6.43% in flexibility per increase of one unit of automaticity (*F*_(__1_,_184__)_ = 83.835; *p* < 0.001; see Figure 4). There was no significant effect of the order of presentation (*F*_(__1_,_82__)_ = 0.238; *p* = 0.627) or habit tendency (*F*_(__1_,_82__)_ = 0.043; *p* = 0.836) on flexibility.

### Convergent Thinking: Candle Problem and Puzzles

The number of participants who solved each problem-solving task is shown in Table 2. In all types of problems (Tasks III and IV), the average score on the SOAT was higher for problem-solvers than for non-solvers (see Table 3).

**TABLE 3:** SOAT score for solvers and non-solvers in Tasks III and IV.

Task	SOAT score solvers	SOAT score non-solvers
Candle Problem	69.83 ± 17.57	64.39 ± 23.03
Pyramid puzzle	74.53 ± 17.50	64.44 ± 18.88
Cube puzzle	72.44 ± 18.39	66.35 ± 19.05

*Score on the SOAT (mean ± SD) for participants who solved or did not solve each of the problem-solving Tasks III and IV.*

*SOAT = Slips-of-Action Task.*

**FIGURE 5:**
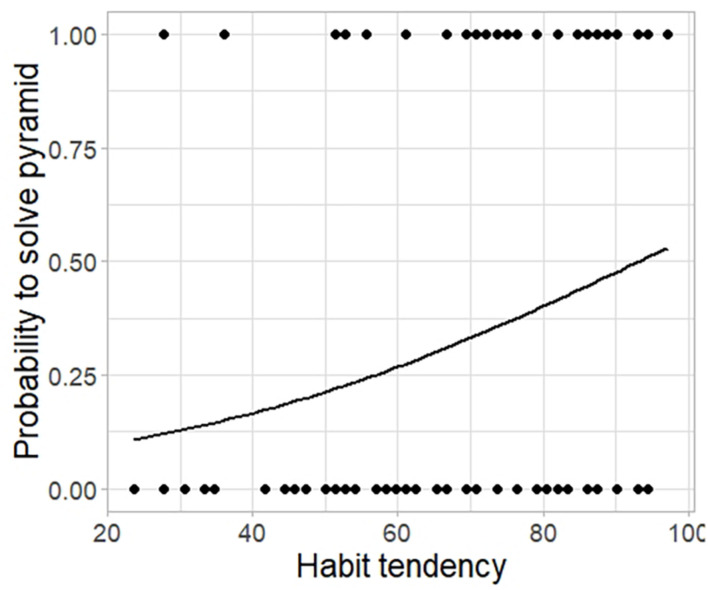
Probability to solve the pyramid puzzle as a function of habit tendency. Probability to solve (binomial response: yes = 1; no = 0) the pyramid puzzle as a function of habit tendency (measured by the SOAT; range: 0–100). A high SOAT score indicates low habit-tendency.

To further test for this, for each type of problem separately, we fitted a generalized (binomial) linear mixed-effects model to the binary outcome variable *solved* as a function of *habit*, with *participant* as a random effect (see Models 4a,b,c of Supplementary Table 2). Habit tendency was a significant predictor for solving success in the pyramid puzzle (*F*_(__1_,_82__)_ = 4.813; *p* = 0.028; Model 4a; see Figure 5): the probability to solve this puzzle was 1% higher per unit of increase in the SOAT score for participants who were less prone to habits. This was not the case for the cube puzzle (*F*_(__1_,_82__)_ = 1.458; *p* = 0.227; Model 4b) or the Candle Problem (*F*_(__1_,_66__)_ = 1.895; *p* = 0.169; Model 4c). The *t*-tests provided similar results as the binomial models, revealing significant differences in habit tendency between solvers and non-solvers for the pyramid puzzle (*t* = 3.077; *df* = 64.133; *p* = 0.003) and for the cube puzzle (*t* = 2.356; *df* = 30.034; *p* = 0.022), but not for the Candle Problem (*t* = 0.558; *df* = 15.758; *p* = 0.584; see Supplementary Table 4).

**FIGURE 6:**
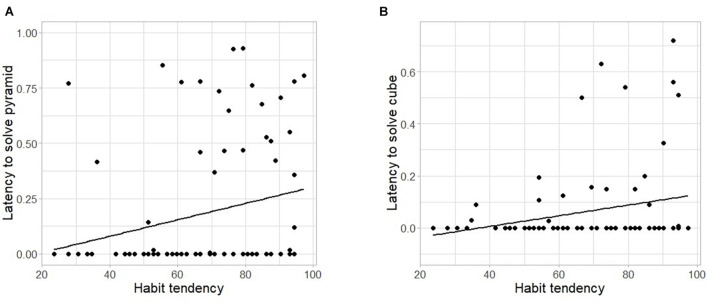
Latency to solve the pyramid and the cube puzzles as a function of habit tendency. Latency (range: 0–1) to solve **(A)** the pyramid puzzle and **(B)** the cube puzzle as a function of habit tendency (measured by the SOAT; range: 0–100). Note that (1) due to the formula applied, higher values of latency correspond to participants who needed less time to solve the task, (2) the Y-axis limit on panel **(B)** was adjusted so that all data points could be plotted, and (3) a high SOAT score indicates low habit-tendency.

Then, for each type of problem separately, we fitted a linear mixed-effects model to the numeric outcome variable *latency* as a function of *habit*, with *participant* as a random effect (see Models 5a,b,c of Supplementary Table 2). In the pyramid puzzle, there was a significant positive relationship between solving speed and SOAT score, with more habit prone individuals generally taking longer to solve this task (solving speed increased by 4.4% per increase in one unit of the SOAT score; *F*_(__1_,_82__)_ = 4.694; *p* = 0.033; Model 5a; see Figure 6A). We found the same relationship for the cube puzzle, with a change in 2.6% per increase in one unit of the SOAT score (*F*_(__1_,_82__)_ = 5.326; *p* = 0.023; Model 5b; see Figure 6B). For the Candle Problem, we did not find a statistically significant relationship between latency and habit tendency (*F*_(__1_,_66__)_ = 0.153; *p* = 0.697; Model 5c).

**FIGURE 7:**
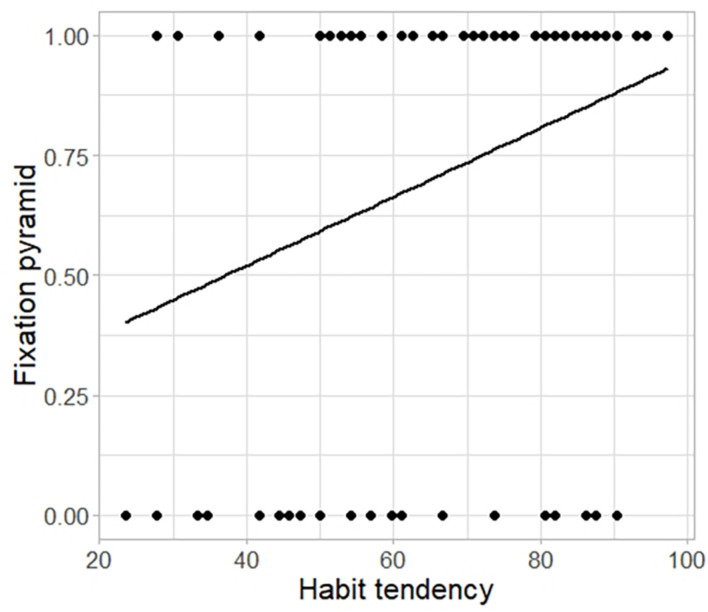
Probability to overcome fixation in the pyramid puzzle as a function of habit tendency. Probability to overcome fixation (range: 0–1) in the pyramid puzzle as a function of habit tendency (measured by the SOAT; range: 0–100). Note that (1) a value of 1 on the y-axis indicates successfully overcoming fixation, as indicated in Table 1, and (2) a high SOAT score indicates low habit-tendency.

Finally, for each type of (puzzle) problem, we fitted a generalized (binomial) linear mixed-effects model to the binary outcome variable *fixation* as a function of *habit*, with *participant* as a random effect (see Models 6a,b of Supplementary Table 2). We found a positive relationship between habit tendency (as measured by the SOAT) and the probability for an individual to have difficulty overcoming fixation in the pyramid puzzle: participants who persisted in using the wrong strategy (i.e., not placing the pieces in the correct position) were 2.5% significantly more prone to habits (*F*_(__1_,_82__)_ = 6.970; *p* = 0.008; see Figure 7; see Model 6a of Supplementary Table 2). However, this was not the case for the cube puzzle (*F*_(__1_,_82__)_ = 1.743; *p* = 0.187; Model 6b).

As a last step, we corrected for multiple comparisons using the Bonferroni method which controls for multiple testing without a loss of power. Observed *p*-values for models 1, 2, and 3 were checked against statistical significance values (α = 0.05) adjusted for the number of predictions tested (*k* = 3), rendering an adjusted value of α = 0.016. Following Bonferroni correction, significance for the correlations of interest (Model 1: automaticity ∼ type of object; Model 2: fluency ∼ type of object; Model 3: flexibility ∼ type of object; flexibility ∼ automaticity) remained unchanged. Significance assessment for the other models (4a–c, 5a–c, and 6a,b), as well as effect sizes and confidence intervals, remained unaffected by the Bonferroni correction.

## Discussion

In this study, we investigated the role of familiarity and habits in mental fixation and divergent thinking by comparing fluency and flexibility scores in the AUT for items used frequently or rarely, and for unknown items, and relating this to subjective automaticity of idea generation. Furthermore, we investigated the relation between individual differences in habit tendency and the aforementioned variables. In support of a fixating effect of habits, we found that frequency of use was a negative predictor of flexibility, and that subjective automaticity was highest for frequent items and had a negative effect on flexibility. At the same time, frequency of use was a positive predictor of fluency. Additionally, we investigated the role of habits on convergent thinking by using a standard task (the Candle Problem) and an exploratory type of task (puzzles). While we found no relationship between individual differences in habit tendency and performance in the AUT or the Candle Problem, we did find that less habit-prone participants were more likely to solve one of the puzzles (the pyramid), and to solve it faster.

Our findings show that frequency of use plays a role in the quality and quantity of ideas evoked: ideas produced for frequently used items were more fluent albeit less flexible. In the case of the unknown items, higher flexibility came at the expense of lower fluency. The higher fluency observed for frequently used items is in line with the notion that habitual S-R associations permit fast retrieval of familiar uses (Benedek et al., 2012; Silvia et al., 2013), which would ultimately result in the production of a larger number of responses. However, the automaticity with which these ideas are generated may take a toll on creativity: these ideas are more conventional, since conceiving unusual ideas requires suppression of well-established S-R associations (Gupta et al., 2012). Indeed, participants reported higher automaticity in thinking of the most common uses for the frequently used items, and subjective automaticity was negatively related to overall flexibility. This finding is in line with our hypothesis that the automatization of ideas about past uses for familiar items could lead to a fixated mindset, thus impeding the ability to think creatively.

Despite finding no evidence in support of an effect of individual differences in habit tendency (SOAT score) on divergent thinking (AUT outcomes), our findings support the notion that strong, automatic S-R associations between an item and its most common use (derived from habitual repetition) can hinder some aspects of creativity. A tendency toward habit can be beneficial or advantageous in certain contexts (e.g., permitting several processes to run in parallel, thus freeing up cognitive resources and enabling efficient responding to demands in everyday life). However, habit tendencies may also lead to rigid, persistent behavior and suboptimal outcomes, particularly when moving past learned behaviors is crucial (Gillan et al., 2011), as is the case in the AUT. Contrary to our prediction, the investigation of the relationship between individual differences in the tendency to rely on habitual (as opposed to goal-directed) control and convergent thinking measures did not find an effect on the Candle Problem or the cube puzzle. Habit tendency was, however, related negatively to performance in the pyramid puzzle: a higher tendency to rely on habits was predictive of slower attainment of success, leading some participants to fail to solve the puzzle within the limited time provided. In fact, forcing participants to make decisions under time pressure may prevent the engagement of flexible control processes and thereby increase reliance on habits (for a review, see Schwabe and Wolf, 2013; Watson and de Wit, 2018). Less habit-prone individuals may have adopted a trial-and-error based searching approach, which, despite being time consuming, provides valuable information about the problem that could help to overcome fixation and arrive at a solution more quickly. The slower performance observed in some individuals on the pyramid puzzle might be caused by a strong fixation effect: it has been shown that some individuals initially tend to revisit potential solutions stored in the memory derived from past experience in similar tasks (Chow et al., 2019). Informal observations made during our study support this interpretation: we often found that, when participants first encountered the puzzle, they tried stacking the pieces in horizontal layers (a fixation-driven configuration), which would impede solving the problem.

Our findings also raise several questions: what caused the different patterns of results with respect to habit tendency for the two convergent thinking tasks? And why did we observe that habits affected one of our convergent thinking measures but not the divergent thinking task? Our study suggests that performance in operant problem-solving tasks (puzzles) is more likely to be influenced by habit tendency than performance in divergent thinking tasks (AUT) or riddle-like tasks (Candle Problem), which are both cognitively more demanding. A fundamental difference in the puzzles compared to the AUT and the Candle Problem was the presentation of the problem’s target elements: while in the puzzles the search process for a solution was constrained by the need to achieve a well-defined goal (Munoz-Rubke et al., 2018), the other two tasks required participants to find a solution “in their head,” and they could not be certain of when exactly the problem was solved. A possible explanation of our findings is that divergent and convergent thinking might be differently affected by habit tendency: while divergent thinking seems to profit from a lower top-down control where attention tends to defocus and flow freely, convergent thinking primarily requires a focus on finding the correct solution (Cropley, 2006) and benefits from a strong degree of goal-directedness and related cognitive control functions, such as working memory and inhibitory control (Akbari Chermahini and Hommel, 2010). Additionally, it is possible that the influence of existing S-R associations might be enhanced in physical contexts: the associated response (solution to the problem) could be more strongly activated when actually manipulating the object (stimulus). This could help to explain the results for the puzzle tasks, where participants were physically engaged in problem-solving. At first glance it might seem that looking at a picture of an object (as in the AUT and the Candle Problem) would not substantially differ from having the actual object in one’s hands (as in the puzzles); however, it is possible that the mental activation of habits is stronger when the object cannot only be seen but also manipulated. Hence, opting for exclusively visual and rather artificial ways of presenting the stimuli (images) might have led to an underestimation of the fixation effects or of the role of habits in the AUT and the Candle Problem. Thus, a promising approach for future investigations would be to design variations of these paradigms with tangible, physical stimuli. On the other hand, since the order in which tasks were presented was not randomized across participants, we cannot discard that the effect of habit tendency on performance in the puzzles can partly be explained by order of presentation. Finally, our relatively low sample size begs consideration of the possibility that the effect of habit tendency on puzzle performance (see Models 4a and 5a,b of Supplementary Table 2) may be an artifact of chance, even more so if we take into account that the final sample size for the Candle Problem was reduced by 16 participants. Given the exploratory nature of this aspect of our study, we therefore emphasize the need for confirmatory follow-up studies with larger sample sizes.

It should also be considered that, when confronted with unknown items in the AUT, participants’ ability to assess the creativity of their responses might not be as accurate for unknown items as for items used frequently, partly because in the former case they may not know the item’s conventional use(s). Therefore, the fact that ideas generated for unknown items were more flexible could partially be explained as a by-product of ambiguity, since some participants may ignore some of the item’s properties (e.g., size, weight, material). Although previous research has shown that fixation can arise due to inaccurate information about an item’s functionality (German and Defeyter, 2000; German et al., 2007), this was not the case in our study, as we found that flexibility was lowest for frequent items and fixation was correspondingly higher for these items, suggesting that our results were mainly driven by S-R associations. Finally, despite our attempt to select items with a similar number of possible functions, we cannot rule out a potential confound between frequency of use and object multi-functionality because we could not objectively determine that all items provided the same number of affordances. Similar future studies could validate and test the generalizability of our results by conducting this task with a wider range of different objects. Moreover, as we did not include a measure of originality in our analysis, we cannot disregard that a different pattern of results could emerge from its study. However, we consider that comparing originality scores across items that differ in their degree of familiarity is a complex issue that should be handled with caution. In our view, the currently existing methods to assess originality may require careful adaptations to fully capture interindividual variations between ideas produced for each type of object in order to ensure that such comparisons remain sensical and reasonable. As this was not part of the scope of our study, we focused on measures for which we had clear predictions (fluency and flexibility). Nonetheless, we (and presumably many researchers in the field of creativity research) look forward to future studies comparing originality between unknown and familiar items.

The present study provides an examination of factors that may impede creative potential and provides evidence for the notion that habits can have a negative impact on creativity by inducing fixation. Furthermore, we showed that individual differences in habit tendency, while seemingly having no measurable effect on divergent thinking (AUT), affected performance on convergent thinking as assessed by puzzles. We also recommend the use of this non-standard methodological tool, especially since it enables physical manipulation of the problem’s target elements. In summary, our study is consistent with the literature showing that bottom-up processes constrain creativity and provides support for the usefulness of self-reported and experimental measures of automaticity and habit in creativity research. Taken together, our findings contribute to a better understanding of the role of habits in creative thinking and open promising future avenues of research in this field.

## Data Availability Statement

The original contributions presented in the study are included in the article/Supplementary Material, further inquiries can be directed to the corresponding author.

## Ethics Statement

The studies involving human participants were reviewed and approved by the University of Amsterdam Faculty of Social and Behavioural Sciences Ethics Review Board (Ref. No.: 2018-CP-8939). The patients/participants provided their written informed consent to participate in this study.

## Author Contributions

All authors designed the study and refined the methodology. PIA conducted the experiments, coded and analyzed the data, and drafted the manuscript. SW and ST contributed to editing the manuscript.

## Conflict of Interest

The authors declare that the research was conducted in the absence of any commercial or financial relationships that could be construed as a potential conflict of interest.

## Publisher’s Note

All claims expressed in this article are solely those of the authors and do not necessarily represent those of their affiliated organizations, or those of the publisher, the editors and the reviewers. Any product that may be evaluated in this article, or claim that may be made by its manufacturer, is not guaranteed or endorsed by the publisher.

## Footnotes

1
https://www.dilemma-games.com
