# Supplementary material for: Can Habits Impede Creativity by Inducing Fixation?
Source: Front Psychol. 2021 Oct 13;12:683024. doi: 10.3389/fpsyg.2021.683024 (PMC8548374; doi:10.3389/fpsyg.2021.683024)
Supplement: Supplementary file 2 [file Data_Sheet_2.PDF]

## Supplementary Materials

## Table 1: List of variables of all tasks presented

| Name of variable | Task(s) | Measures per participant | Type of variable | Range of values | What does it measure? | How was it calculated? |
|---|---|---|---|---|---|---|
| **Participant** | - | 1 | categorical | - | Alphanumeric code to identify participants | Number from 1 to 85 + first letter of participant's name and surname |
| **Order of presentation** | AUT | 1 | numeric ordinal | From 1 to 3 | Order of presentation of items on the AUT | Order 1: freq-rare-unk<br>Order 2: unk-freq-rare<br>Order 3: rare-unk-freq |
| **Type of object** | AUT | 3 | categorical | freq-rare-unk | Type of object on the AUT | Frequent-freq (pencil and cup)<br>Rare (rope and brick)<br>Unknown-unk (whisk and medical instrument) |
| **Fluency** | AUT | 3 | numeric | From 1 to infinity | Number of responses per item per participant | Summing up all responses per item per participant, then averaging between the two items of the same type. |
| **Flexibility** | AUT | 3 | numeric | From 0 to 1 | Number of categories of use cited per participant per item. | Dividing the number of categories cited per participant for an item by the maximum number of categories cited by someone for that same item. Then averaging both scores of the same type of item. |
| **Automaticity** | AUT | 3 | numeric | 5-point Likert scale | Self-report on the degree of automaticity when thinking of the most common use for an item. | Participants were asked to rate how automatically they thought of the most common use for an item, from 1 (not automatically at all) to 5 (very automatically) |

| | | | | | | |
|---|---|---|---|---|---|---|
| **Frequency of use** | AUT | 3 | numeric | 5-point Likert scale | Self-reported frequency of use of the items shown on the AUT | Participants were asked to rate how frequently they personally used each item, from 1 (never) to 5 (daily) |
| **Habit** | SOAT | 1 | numeric | From - 100 to +100 | Participant's habitual vs goal directed control: Difference score to control for variations in responding to valued (go) versus devalued (no-go) trials. | Percentage of responses for valued minus devalued trials |
| **Type of problem** | Candle Problem + pyramid puzzle + cube puzzle | 3 (one for each problem type) | categorical | Candle Problem - pyramid puzzle-cube puzzle | The type of problem used on the problem-solving tasks (III and IV) | - |
| **Solved** | Candle Problem + pyramid puzzle + cube puzzle | 3 (one for each problem type) | binary | 0 or 1 | A participant's ability to solve a problem within the time given | 0 if not solved or gave up<br>1 if solved<br>NA for those who knew the problem |
| **Fixation** | Candle Problem pyramid puzzle + cube puzzle | 2 (1 for the pyramid puzzle, 1 for the cube puzzle) | binary | 0 or 1 | A participant's ability to break away from fixation on a problem-solving task. Overcoming fixation is defined as 'using balls in vertical' for the pyramid puzzle, and 'building cube outside the box' for the cube puzzle. | 0: not overcoming fixation<br>1: overcoming fixation |
| | | | numeric | | | Candle Problem:<br>0 if not solved or gave up |

| | | | | | |
|---|---|---|---|---|---|
| **Latency** | Candle Problem + pyramid puzzle + cube puzzle | 3 (one for each problem type) | From 0 to 1 | Time consumed to solve or give up on a problem-solving task. The maximum time provided is 480 seconds (8 minutes) for the Candle Problem, and 300 seconds (5 minutes) for each of the puzzles. | For solvers = 1 - (time consumed/480)<br>Puzzles:<br>0 if not solved or gave up<br>For solvers = 1 - (time consumed/300) |

**Table 2: Summary of model outputs.** Model syntax: lmer, glmer: type of model applied (lmer = linear mixed effects regression; glmer = generalized linear mixed effects regression); the (~) symbol separates the model response (to the left) from the model predictors (to the right); '(1|participant)': indicates that 'participant' was entered as a random effect; 'family = binomial': a binomial glmer model was applied when response variable was binary. Note: for Models 1, 2 and 3, 'type of object = unknown' was taken as a reference level. Legend: (*) Significant *p*-values at $\alpha = 0.05$ (shown in bold). Abbreviations: type obj = type of object (in the AUT); Pyr = pyramid; Estim. = estimate; Eff. size = effect size; SE = standard error; Low. CI = lower confidence interval at 95%; Up. CI = upper confidence interval at 95%.

| Model syntax | Response | Predictor(s) | Estim. | Eff. size | SE | *p*-value | Low. CI | Up. CI |
|---|---|---|---|---|---|---|---|---|
| Model 1 = lmer (automaticity ~ type of object + habit + (1\|participant)) | Automaticity | Type obj rare | 1.348 | 153.15 (+53%) | 0.119 | **< 0.001*** | 1.113 | 1.581 |
| | | Type obj freq | 2.341 | 192.31 (+92%) | 0.119 | **< 0.001*** | 2.107 | 2.575 |
| | | Habit | 0 | 0 | 0.002 | 0.9 | -0.005 | 0.006 |
| Model 2 = lmer (fluency ~ type of object + order of presentation + habit + automaticity + (1\|participant)) | Fluency | Type obj rare | 1.976 | 129.3 (+30%) | 0.195 | **< 0.001*** | 1.598 | 2.389 |
| | | Type obj freq | 1.905 | 128.8 (+29%) | 0.195 | **< 0.001*** | 1.501 | 2.291 |
| | | Order of presentation | -0.233 | 96.5 (-3.5%) | 0.308 | 0.452 | -0.832 | 0.372 |
| | | Habit | 0 | 0 | 0.013 | 0.637 | -0.020 | 0.032 |
| | | Automaticity | -0.142 | 97.9 (-2.1%) | 0.013 | 0.273 | -0.394 | 0.108 |
| Model 3 = lmer (flexibility ~ type of object + order of presentation + habit + automaticity + (1\|participant) | Flexibility | Type obj rare | -0.139 | 78.3 (-21.7%) | 0.016 | **< 0.001*** | -0.171 | -0.107 |
| | | Type obj freq | -0.175 | 72.7 (-27.3%) | 0.022 | **< 0.001*** | -0.217 | -0.132 |
| | | Order of presentation | -0.007 | 98.5 (-1.5%) | 0.015 | 0.627 | -0.037 | 0.022 |
| | | Habit | 0 | 0 | 0 | 0.836 | -0.001 | 0.001 |
| | | Automaticity | -0.047 | 93.6 (-6.4%) | 0.005 | **< 0.001*** | -0.057 | -0.037 |
| Model 4 = glmer (solved ~ habit + (1\|participant), family = binomial) | Solved | Model 4a (Pyr): Habit | 0.030 | 101.06 (+1%) | 0.013 | **0.028*** | 0.006 | 0.063 |
| | | Model 4b (Cube): Habit | 0.017 | 99.3 (-0.7%) | 0.015 | 0.227 | -0.009 | 0.056 |
| | | Model 4c (Candle): Habit | 0.023 | 99.1 (-0.9%) | 0.017 | 0.169 | -0.010 | 0.069 |
| Model 5 = lmer (latency ~ habit + (1\|participant)) | Latency | Model 5a (Pyr): Habit | 0.003 | 104.4 (+4%) | 0.001 | **0.033*** | 0 | 0.007 |
| | | Model 5b (Cube): Habit | 0.002 | 102.63 (+2%) | 0 | **0.023*** | 0 | 0.004 |
| | | Model 5c (Candle): Habit | 0 | 0 | 0.001 | 0.697 | -0.004 | 0.003 |
| Model 6 = glmer (fixation ~ habit + (1\|participant), family = binomial) | Fixation | Model 6a (Pyr): Habit | 0.036 | 102.48 (+2%) | 0.013 | **0.008*** | 0.012 | 0.070 |
| | | Model 6b (Cube): Habit | 0.016 | 96.9 (-3.1%) | 0.012 | 0.187 | -0.007 | 0.042 |

**Table S3: Survey conducted prior to study to establish the frequency of use categories presented in the AUT.** 30 volunteers were asked to rate on a 10-objects list how often they used each one of them by using a 5-point Likert scale (i.e., 1 = never; 5 = every day). Outcome responses were divided into 3 groups as follows: frequently used (cup, elastic rubber band, pencil, ring), rarely used (brick, needle, rope), never used (bamboo whisk, medical instrument, pipe). Two objects from each group category were picked at random, constituting the target stimuli presented in the AUT.

| | **1** | **2** | **3** | **4** | **5** |
|---|---|---|---|---|---|
| Bamboo whisk | | | | | |
| Brick | | | | | |
| Cup | | | | | |
| Elastic rubber band | | | | | |
| Medical instrument | | | | | |
| Needle | | | | | |
| Pencil | | | | | |
| Pipe | | | | | |
| Rope | | | | | |
| Ring | | | | | |

**Table S4: Independent *t*-tests for habit differences between solvers and non-solvers in tasks III (Candle Problem) and IV (Pyramid and Cube puzzles).** Legend: (*) Significant *p*-values at $\alpha = 0.05$ (shown in bold). Abbreviations: S = (SOAT score for) solvers; NS = (SOAT score for) non-solvers; N = sample size; SD = standard deviation; CI = confidence intervals at 95%.

| **Task** | **N** | **Mean ± SD** | ***p*-value** | ***t*-value** | **Lower CI** | **Upper CI** |
|---|---|---|---|---|---|---|
| Candle Problem | S: 56<br>NS: 13 | S: 69.83 ± 17.57<br>NS: 64.39 ± 23.03 | 0.584 | 0.558 | -10.145 | 17.368 |
| Pyramid puzzle | S: 28<br>NS: 50 | S: 74.53 ± 17.50<br>NS: 64.44 ± 18.88 | **0.003*** | 3.077 | 4.706 | 22.148 |
| Cube puzzle | S: 20<br>NS: 63 | S: 72.44 ± 18.39<br>NS: 66.35 ± 19.05 | **0.022*** | 2.356 | 1.238 | 5.345 |
